# A Structured Assessment to Decrease the Amount of Inconclusive Endometrial Biopsies in Women with Postmenopausal Bleeding

**DOI:** 10.1155/2016/3039261

**Published:** 2016-03-13

**Authors:** M. C. Breijer, N. C. M. Visser, N. van Hanegem, A. A. van der Wurff, B. C. Opmeer, H. C. van Doorn, B. W. J. Mol, J. M. A. Pijnenborg, A. Timmermans

**Affiliations:** ^1^Department of Obstetrics and Gynecology, Erasmus MC Cancer Institute, Postbus 2040, 3000 CA Rotterdam, Netherlands; ^2^Department of Obstetrics and Gynecology, Albert Schweitzer Hospital, Postbus 444, 3300 AK Dordrecht, Netherlands; ^3^Department of Pathology, Radboud University Medical Center, Postbus 9101, 6500 HB Nijmegen, Netherlands; ^4^Department of Obstetrics and Gynecology, Academic Medical Center, Postbus 22660, 1100 DD Amsterdam, Netherlands; ^5^Department of Obstetrics and Gynecology, Maastricht University Medical Center, Postbus 5800, 6202 AZ Maastricht, Netherlands; ^6^Department of Pathology, St. Elisabeth Hospital, Hilvarenbeekseweg 60, 5022 GC Tilburg, Netherlands; ^7^Clinical Research Unit, Academic Medical Center, Postbus 22660, 1100 DD Amsterdam, Netherlands; ^8^The Robinson Research Institute, School of Paediatrics and Reproductive Health, University of Adelaide and South Australian Health and Medical Research Institute, Adelaide, SA 5000, Australia; ^9^Department of Obstetrics and Gynecology, TweeSteden Hospital, Dr. Deelenlaan 5, 5042 AD Tilburg, Netherlands

## Abstract

*Objective.* To determine whether structured assessment of outpatient endometrial biopsies decreases the number of inconclusive samples.* Design.* Retrospective cohort study.* Setting.* Single hospital pathology laboratory.* Population.* Endometrial biopsy samples of 66 women with postmenopausal bleeding, collected during the usual diagnostic work-up and assessed as insufficient for a reliable histological diagnosis.* Methods.* Endometrial biopsy samples were requested from the pathology laboratories. The retrieved samples were systematically reassessed by a single pathologist specialized in gynecology.* Main Outcome Measure.* Disagreement between initial assessment and conclusion after structured reassessment.* Results.* We retrieved 36 of 66 endometrial biopsy samples from six different pathology laboratories. Structured reassessment of the retrieved samples by a single pathologist specialized in gynecology did not change the conclusion in 35 of the 36 samples. The remaining sample contained a large amount of endometrial tissue and the diagnosis at reassessment was endometrial hyperplasia without atypia. All other samples contained insufficient material for a reliable diagnosis.* Conclusion.* A structured reassessment of endometrial biopsies samples, which were classified as inconclusive due to insufficient material, did not change the conclusion. Although it might be helpful for pathologists to have diagnostic criteria for adequacy and/or inadequacy of an endometrial biopsy sample, the gain in efficiency is likely to be small.

## 1. Introduction

Patients with postmenopausal bleeding (PMB) are at risk for endometrial carcinoma and therefore PMB warrants further investigation. Histological endometrial assessment is indicated when a patient presents with postmenopausal bleeding and an increased endometrial thickness on transvaginal sonography (TVS) [1, 2]. Outpatient endometrial biopsy is the least invasive technique to obtain material for histological assessment. Endometrial biopsies have a very high sensitivity for diagnosing an endometrial (pre)malignancy in postmenopausal women (95%) [3]. Furthermore, performing an endometrial biopsy in women with PMB and an increased endometrial thickness is the most cost-effective strategy [4].

Yet, 7–68% of outpatient endometrial biopsy samples are inconclusive because the amount of tissue obtained is insufficient for a reliable histopathological diagnosis [5–8]. In such cases, a more invasive hysteroscopy or dilatation and curettage (D&C) is necessary in order to rule out endometrial carcinoma or atypical hyperplasia, which is present in 6% of these women [7]. The high failure rate due to inconclusive endometrial biopsies might affect the cost-effectiveness of the diagnostic work-up.

Among pathologists considerable disagreement exists about what constitutes an adequate endometrial biopsy sample. A questionnaire sent to all members of the British Association of Gynecological Pathologists and participants in the National Gynecological Pathology External Quality Assessment Scheme in the UK revealed that 88.5% of the respondents would think that criteria for adequacy and/or inadequacy would be useful [9]. Such guidelines are at present not available. This results in a diagnosis that is influenced by subjectivity, with a high interobserver variability between pathologists.

The objective of the current study was to determine whether structured assessment of outpatient endometrial biopsy samples with strict criteria decreases the amount of inconclusive samples due to insufficient material.

## 2. Material and Methods

**Figure 1:**
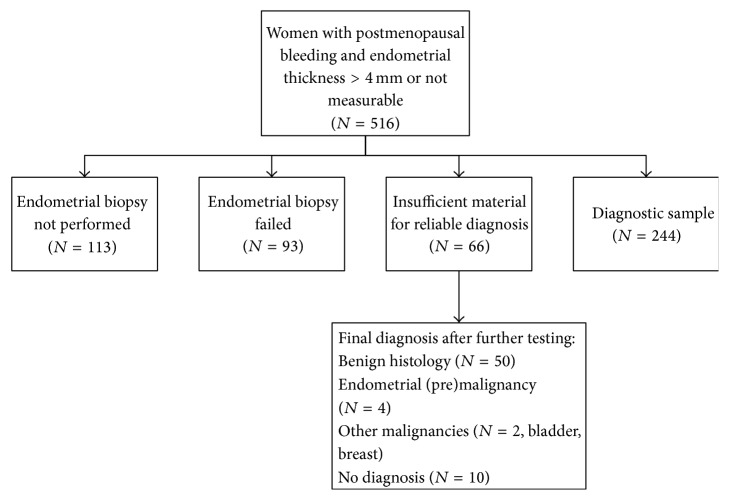
Flowchart of women with postmenopausal bleeding included in the original prospective cohort study.

Material collected during the diagnostic work-up of women participating in a previous prospective cohort study on PMB was used for the present study [7]. Details of the cohort are presented in the original publication. In short, data on all women presenting with PMB in seven teaching hospitals and one university hospital in Netherlands, between January 2001 and June 2003, were prospectively collected to evaluate the diagnostic work-up in women with PMB in Netherlands [7]. During the study period, 516 women with PMB were seen in the outpatient clinic. If the endometrial thickness measured more than 4 mm and in case of an unclear endometrial thickness, an endometrial biopsy was indicated. In 403 of those women, an outpatient endometrial biopsy was performed. In this study there was an inconclusive endometrial biopsy sample due to insufficient tissue in 66 of the 403 women. All 66 women were subsequently evaluated with hysteroscopy or D&C and in 4 women (6%) an endometrial (pre)malignancy was diagnosed. The 66 cases with an inconclusive endometrial biopsy in the prospective cohort were used for the current study (Figure 1).

The endometrial biopsy samples were requested from the pathology laboratories of the eight hospitals. Based on two reports on the assessment of endometrial biopsy samples, we proposed items for a structured assessment (Table 1) [9, 10]. The material was systematically scored on the items “estimated amount of material” and “quality of material.” The endometrial biopsy samples were systematically reassessed by a single pathologist specialized in gynecology (AW). The pathologist knew that it was a reassessment of previously nondiagnostic endometrial biopsy samples but was blinded to the final diagnosis in the original cohort study. If the quality of the slides was decreased due to their age, new HE stained slides were made; paraffin blocks were always at hand. The study was reviewed and approved by the institutional review board.

### 2.1. Statistical Analysis

Statistical analysis was performed with the PASW statistics 18.0 package (SPSS, Inc., Somers, NY, USA). Depending on the distribution, continuous variables were presented as mean ± standard deviation or as median and interquartile range, and differences between groups tested with an independent samples *t*-test or a Mann-Whitney *U* test. Categorical data were presented as *N* (%) and groups compared using a Chi-square test.

## 3. Results

We were able to retrieve endometrial biopsy samples from 36 (55%) women from six different pathology laboratories. Two hospitals did not respond to our request to send material. Pathology laboratories that did not respond to the initial request were contacted by phone once. Afterwards, no further efforts were made to obtain the samples.

In endometrial biopsy samples from 29 (80.5%) of the 36 women, no endometrial tissue was found. The pathologist described just mucus, blood, or superficial columnar epithelium. In endometrial biopsy samples from six (16.7%) women, superficial endometrium was found, with only one very small strip of endometrial stroma, and therefore these samples were also inconclusive. In one (2.89%) endometrial biopsy sample, the amount of endometrium present was sufficient for assessment, and the diagnosis simple endometrial hyperplasia without atypia was established on this sample. The reassessment for all 36 women with the macroscopic description is reported in Table 2.

Characteristics of women from whom no material was received were compared to those of women with reassessed samples; no differences were found (Table 3). All samples of women that were initially inconclusive but diagnosed with endometrial (pre)malignancy at subsequent testing were available for reassessment.

## 4. Discussion

A structured reassessment of endometrial biopsy samples that were initially classified as inconclusive due to insufficient material did not change the conclusion in the majority of cases. To our knowledge, there are no previous reports on the attempt to increase the diagnostic efficiency of outpatient endometrial biopsy by structured assessment.

In women with PMB and an endometrial thickness more than 4 mm or an unclear endometrial thickness and an inconclusive outpatient endometrial biopsy, an endometrial (pre)malignancy is found in 6%. Therefore, these women cannot be reassured without further, more invasive, diagnostics [7].

Reviewing hospital protocols revealed that a protocol on standardized sampling methods was not available in any hospital, let alone the recommendations on using a tenaculum, entering the uterine cavity more than once, or the use of analgesia in painful procedures.

The strength of our study is that all endometrial biopsy samples were assessed by one pathologist specialized in gynecology who was blinded to the definite diagnosis. Yet, our study is limited by the fact that only 55% of the requested endometrial biopsy samples were retrieves. It is however unlikely that a complete response would have changed our conclusions. Only one of the 36 available endometrial biopsy samples was considered diagnostic at reassessment within this selection, and second, all samples of women that were initially inconclusive but diagnosed with endometrial (pre)malignancy at subsequent testing were available for reassessment. Characteristics of women from whom no material was received were compared to those of women with reassessed samples; no differences were found. Another limitation of our study is that the pathologist performing the reassessment knew that the endometrial biopsy samples were initially assessed as inconclusive, which potentially leads to a biased interpretation at reassessment. Performing a prospective cohort study where all endometrial biopsy samples are assessed with structured criteria during a certain period could solve this. The amount of insufficient samples could be compared to the amount of insufficient samples in a previous period without structured pathology assessment.

In 2005 Phillips and McCluggage reported on a questionnaire among 61 pathologists in the UK. Most respondents felt that it would be useful if criteria for adequacy and/or inadequacy were proposed [9]. Similar criteria are already used for the evaluation of specimen adequacy in fine needle aspiration of thyroid nodules [11].

In the cost-effectiveness analysis by Clark et al., the failure rate due to inconclusive endometrial biopsy samples was 12% (95% CI 0.09–0.15) based on a systematic review [4]. In our cohort study on PMB, 66 out of 403 endometrial biopsies were inconclusive (16.4%). Other studies report a failure rate of 7–68% [5–8]. The high failure rate of endometrial biopsies might have consequences for the cost-effectiveness of the technique.

## 5. Conclusions

Our findings suggest that although it might be helpful for pathologists to have diagnostic criteria for adequacy and/or inadequacy of an endometrial biopsy sample, the gain in efficiency is likely to be small. We therefore think that, to increase the effectiveness of outpatient endometrial biopsies, the physician performing an outpatient endometrial biopsy should try to obtain as much material as possible to minimize the chance of a failed biopsy. Further research should focus on the best way to achieve this.

## Figures and Tables

**Table 1:** Items for assessment of the adequacy of an endometrial biopsy sample.

Items	
Estimated amount of material	No material
	<0.5 cm^2^
	0.5–1.0 cm^2^
	1.0–2.0 cm^2^
	>2.0 cm^2^

Quality of material	No endometrial tissue
	Only superficial endometrial tissue
	A large amount of endometrial tissue

**Table 2:** Reassessment of endometrial samples.

Patient	Macroscopic	Amount (cm^2^)	Quality	Diagnosis
1	5 mL mucus	<0.5	No endometrial tissue	No diagnosis
2	Tissue 15 × 3 mm	<0.5	No endometrial tissue	No diagnosis
3	0.5 mL tissue	<0.5	No endometrial tissue	No diagnosis
4	Just mucus and blood	<0.5	No endometrial tissue	No diagnosis
5	1 mL tissue	<0.5	No endometrial tissue	No diagnosis
6	Just mucus and blood	<0.5	No endometrial tissue	No diagnosis
7	Some tissue 1 to 4 mm	<0.5	Superficial endometrial tissue	No diagnosis
8	Some white tissue	<0.5	No endometrial tissue	No diagnosis
9	Red/brown mucinous tissue	<0.5	No endometrial tissue	No diagnosis
10	Very little mucinous tissue	<0.5	No endometrial tissue	No diagnosis
11	Very little grey/red material	<0.5	No endometrial tissue	No diagnosis
12	Some grey/red tissue fragments maximum 3 cm	>2.0	Large amount of endometrial tissue	Hyperplasia without atypia
13	Little grey/brown material	<0.5	No endometrial tissue	No diagnosis
14	Hardly any material	<0.5	No endometrial tissue	No diagnosis
15	Very little grey/brown material	<0.5	No endometrial tissue	No diagnosis
16	1 mL mucus	<0.5	No endometrial tissue	No diagnosis
17	0.5 mL mucus	<0.5	No endometrial tissue	No diagnosis
18	<0.5 mL mucus	<0.5	No endometrial tissue	No diagnosis
19	Some tissue fragments <0.5 mm	<0.5	No endometrial tissue	No diagnosis
20	0.5 mL mucinous bloody material	<0.5	No endometrial tissue	No diagnosis
21	15 mL mucinous material	<0.5	No endometrial tissue	No diagnosis
22	<0.25 mL mucus	<0.5	No endometrial tissue	No diagnosis
23	Some mucinous fragments	<0.5	Superficial endometrial tissue	No diagnosis
24	0.5 mL mucinous bloody material	<0.5	Superficial endometrial tissue	No diagnosis
25	0.5 mL material	<0.5	Superficial endometrial tissue	No diagnosis
26	Some very small fragments	<0.5	No endometrial tissue	No diagnosis
27	0.5 mL mucinous material	<0.5	No endometrial tissue	No diagnosis
28	<0.5 mL mucinous material	<0.5	No endometrial tissue	No diagnosis
29	Three mucinous fragments 1-2 mm	<0.5	Superficial endometrial tissue	No diagnosis
30	0.5 mL mucinous material	<0.5	No endometrial tissue	No diagnosis
31	<0.25 mL mucus	<0.5	No endometrial tissue	No diagnosis
32	0.25 mL mucus	<0.5	No endometrial tissue	No diagnosis
33	<0.2 mL material	<0.5	No endometrial tissue	No diagnosis
34	Just mucus	<0.5	No endometrial tissue	No diagnosis
35	0.2 mL material	<0.5	Superficial endometrial tissue	No diagnosis
36	Mucinous material	<0.5	No endometrial tissue	No diagnosis

**Table 3:** Characteristics of women with a nondiagnostic sample, retrieved samples versus not retrieved samples.

Characteristic	Material retrieved (*N* = 36)	Material not retrieved (*N* = 30)	*P* value
Age (years)^a^	62.5 ± 8.7	61.9 ± 10.0	*P* = 0.39
Body mass index (kg/m^2^)^a^	29.1 ± 6.35	27.5 ± 4.84	*P* = 0.53
Endometrial thickness (mm)^b^	7.5 (6.0–13.5)	7.0 (6.0–10.5)	*P* = 0.42
Hormone therapy use *n* (%)^c^	7 (19.4%)	7 (23.3%)	*P* = 0.89
Nulliparity *n* (%)^c^	2 (5.6%)	3 (10%)	*P* = 0.69
Endometrial (pre)malignancy *n* (%)^c^	4 (11.1%)	0	*P* = 0.03

^a^Independent samples *t*-test, mean ± SD.

^b^Mann-Whitney *U* test, median IQR.

^c^Chi-square.
